# An Unusual Presentation of Juvenile Polymyositis in an Adolescent Girl

**DOI:** 10.7759/cureus.33249

**Published:** 2023-01-02

**Authors:** Rushi Patel, Urvi Zala, Joy Chaudhari, Viraj Panchal, Abhi Shah

**Affiliations:** 1 Medicine, Smt. N.H.L. Municipal Medical College, Ahmedabad, IND

**Keywords:** drooling of saliva, proximal muscle weakness, oropharyngeal dysphagia, juvenile polymyositis, generalized body swelling

## Abstract

Juvenile idiopathic inflammatory myopathies (JIIMs) are a group of diverse, systemic autoimmune diseases that manifest in childhood and are characterized by weakness and chronic inflammation of skeletal muscles. One of the relatively rare variants of JIIMs is juvenile polymyositis (JPM). JPM patients present with proximal and distal muscle weakness, gait instability with falls, muscle pain and tenderness, and high levels of creatine kinase (CK) during adolescence. There are currently few people being diagnosed with JPM, which raises the question of whether or not it is a distinct disease. We discuss the case of a 13-year-old girl who presented to the hospital with generalized body swelling and difficulty swallowing solid food. She also had drooling of saliva during the presentation and a history of difficulty climbing up and down the stairs for three months. Her extensive laboratory workup showed a positive antinuclear antibody (ANA) test and increased muscle enzyme. A muscle biopsy was ordered, and she was diagnosed with JPM. Such a unique presentation has rarely been reported in the pediatric literature.

This case report outlines an unusual JPM presentation that could help clinicians identify the condition and start treatment as soon as possible to minimize complications.

## Introduction

Juvenile idiopathic inflammatory myopathies (JIIMs) are a rare category of systemic autoimmune illnesses defined by persistent skeletal muscle inflammation of unknown origin that manifests itself at the age of less than 18 years [1].

There are two significant subsets: juvenile dermatomyositis (JDM) and juvenile polymyositis (JPM). JDM is the most common clinical subtype, accounting for 80% of all JIIMs while JPM accounts for 2-8% of all JIIMs [2,3]. Clinically, JDM presents with muscle and skin involvement. The skin involvement typically includes a heliotropic rash, which often presents as erythematous discoloration of the eyelids, and Gottron’s papules, which are raised erythematous rashes over the knuckles. On biopsy, there is a peculiar perimysial inflammation and atrophy with surrounding helper T cells (CD4+). JPM, on the other hand, presents with symmetric proximal muscle weakness and is characterized by predominant endomysial inflammation with cytotoxic T cells (CD8+).

Usually, the initial presenting complaint in these myopathies is muscle weakness; rarely do they present with generalized body swelling and dysphagia, even rarer considering JPM, it is a challenge to establish the diagnosis when a young patient presents with such atypical initial symptoms. We report a rare case of a 13-year-old female patient who presents with generalized body swelling, dysphasia, and several months of symmetric proximal muscle weakness. Further assessment with a laboratory workup and a muscle biopsy revealed the diagnosis of JPM. There have been very few instances reported in the pediatric literature with widespread body swelling as a presenting complaint of JPM [4].

## Case presentation

**Figure 1:**
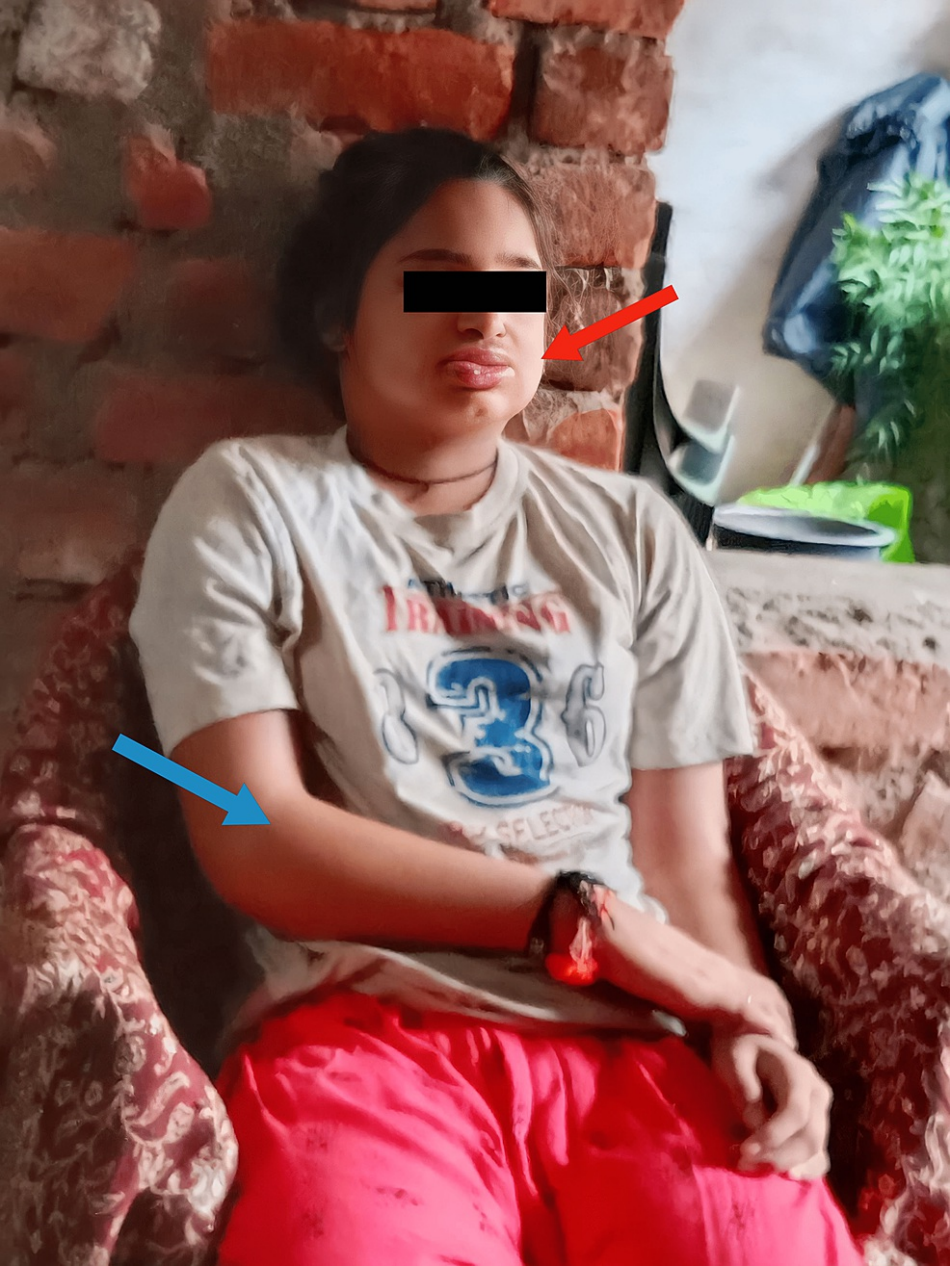
Generalized body edema Facial edema, including the lips (red arrow), and upper extremity edema (blue arrow)

A 13-year-old girl presented to the hospital with a five-day history of progressive generalized body swelling, as shown in Figure 1, and severe dysphagia.

The swelling was first noted on the face and gradually progressed, involving the upper and lower extremities. The swelling was pitting in nature, and there was no associated erythema or tenderness on palpation. Drooling of saliva was present at the time of hospital admission, necessitating the use of a nasogastric tube. On further questioning, the patient’s parents revealed that she also had difficulty walking and climbing up and down the stairs for three months, along with intermittent low-grade fever at night. The patient denied any recent history of falls, shortness of breath, change in color of urine, recent infection, rash, previous history of similar episodes, or recent travel. She had no significant family history of muscular dystrophy or systemic lupus erythematosus.

On examination, she was afebrile with normal vital signs. A neurological examination showed normal mental function. Bilateral proximal muscle weakness of the lower limb was noted, which was symmetric in nature. The patient was unable to stand up from a lying down position and could not walk without support. Muscle power was graded 2 and 3 in the proximal and distal lower limb muscles, respectively, according to the Medical Research Council (MRC) scale. Deep tendon reflexes were elicited, and the sensation was intact.

Laboratory workup showed microcytic and mildly hypochromic anemia; the antinuclear antibody (ANA) test was positive; and creatinine kinase, a muscle enzyme, was significantly increased. A biopsy of the muscle revealed mild endomysial lymphocytic inflammatory infiltrates with muscle fiber size variation and foci of regenerative changes, which clinched the diagnosis of JPM. Her extensive laboratory workup is shown in Table 1.

**Table 1:** Laboratory workup of the patient during hospitalization RDW-CV: red-cell distribution width-coefficient of Variation; ANA: antinuclear antibody; RA factor: rheumatoid arthritis factor; Anti-Ds DNA: anti-double-stranded DNA; TSH: thyroid-stimulating hormone

Laboratory Workup		
Investigations	Patient’s value	Reference value
Hemoglobin (g/dl)	9.7	11.4 - 15.4
RDW-CV (%)	14.8	11.6 - 14.6
WBC count (cells/mm3)	10960	4000 - 11000
Neutrophils (%)	72	40 - 70
Lymphocytes (%)	18	28 - 48
Eosinophil (%)	1	1 - 6
Monocytes (%)	9	2 - 10
Basophils (%)	0	0 - 1
Platelet count (cells/mm3)	294400	150000 - 450000
Serum creatinine (mg/dl)	0.35	0.6 - 1.2
SGPT (IU/L)	77.29	0 - 49
Total protein (g/dl)	5.31	6 - 8
Albumin (g/dl)	3.42	3.4 - 5
Globulin (g/dl)	1.89	2 - 4.1
ANA	Positive	Positive
RA factor (IU/mL)	4.56	0-20
Anti-Ds DNA	Negative	Negative
Creatinine phosphokinase (U/L)	2382	24 - 195
TSH (µIU/mL)	3.764	0.7 - 6.40
C-reactive protein (mg/L)	3.85	0 - 6
Antistreptolysin O (IU/mL)	113.69	0 - 200
Jo-1 Antibody	Negative	Negative

**Figure 2:**
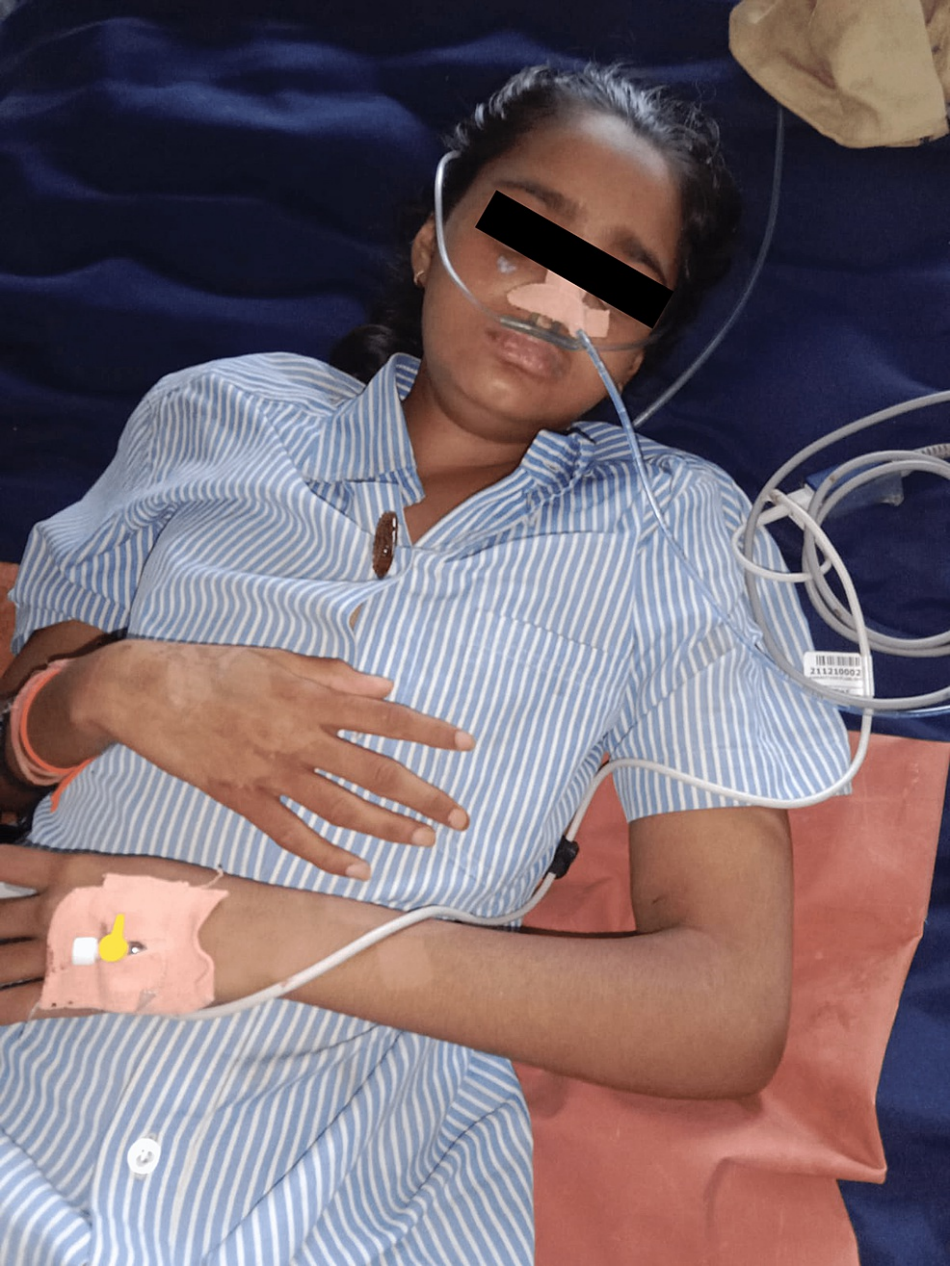
Resolution of edema Resolution of the edema noted around the face, lips, and upper extremities

The patient was treated with intravenous methylprednisolone 1 gram/day for three days, followed by oral prednisolone 40 milligrams/day for the next 10 days. There was a marked improvement in edema as shown in Figure 2, and the patient felt better.

She subsequently developed pneumonia, presumably due to aspiration of the oropharyngeal microbes as a result of dysphagia. She developed a fever of 101° F, accompanied by crepitations in the right middle and lower lobes. There was no associated cough or dyspnea. The chest X-ray showed consolidation in the right middle and lower lobes. She was treated empirically with cefoperazone sulbactam 1.5 grams twice daily for five days via I.V. infusion, which led to the gradual resolution of pneumonia. Based on a rheumatologist consultation, she was referred to a tertiary care hospital for intravenous immunoglobulin treatment. Once the patient’s condition stabilized, she was discharged with regular follow-up arranged. After a few days, the patient died during her sleep at home. She was immediately brought to the hospital, but the exact cause of death could not be determined.

## Discussion

JPM and JDM are rare autoimmune myopathies in childhood. Proximal muscle weakness is a defining feature of both disorders; however, skin involvement is typically absent in JPM. The unique feature of our patient was generalized body swelling along with severe dysphagia. In the pediatric literature, the initial presentation of JPM patients with generalized body swelling has been described before [4]. However, the presentation of patients with accompanying symptoms of dysphagia has not been documented yet. Unlike polymyositis, dermatomyositis has been associated with anasarca [5-8].

Myositis was suspected because of the patient’s symmetric weakness and inability to stand up from a lying down position. A positive ANA profile and an elevated muscle enzyme on the laboratory workup further corroborated the suspicion of inflammatory myositis. A muscle biopsy was performed, showing an endomysial lymphocytic inflammation infiltrate, confirming the diagnosis of JPM.

The precise mechanism of polymyositis-induced generalized edema is unknown. Autoantibodies are thought to play a significant role in pathogenesis. Because polymyositis-dermatomyositis is a type of immune complex vasculitis characterized by endothelial damage due to the complement C5b-9 attack complex, it has been proposed that as a result of diffuse and widespread capillary endothelial damage, there is an increase in capillary permeability in muscles, resulting in generalized swelling [9]. These instances may initially be misinterpreted as having nephritic syndrome at the time of presentation [6,7].

The possible reason for severe dysphagia in our patient could be that oropharyngeal swallowing is aided by their many striated muscles, which are innervated by the lower cranial nerves bilaterally. Any systemic condition affecting the striated muscles has the potential to likewise have an impact on the oropharyngeal muscles. As a result, clinical and electrophysiological involvement of the oropharyngeal muscles in polymyositis dysphagia is anticipated [10]. Kagen LJ et al. state that dysphagia may arise due to myogenic atrophy of the cricopharyngeus muscle, weakening of the gullet entry wall, widespread weakness of the superior constrictor muscles of the pharynx, and inflammatory cell invasion of the muscular connective tissue [11].

JPM is a rare condition, and its presentation with atypical symptoms makes it even more challenging to identify. Two such unusual symptoms are generalized body edema and dysphagia. There have been reported cases of JPM with a presenting complaint of proximal muscle weakness, but to our knowledge, there has not been a reported case with an initial presentation of body swelling along with saliva dribbling and difficulty swallowing. The patient presented in this case report can help clinicians diagnose JPM in children and provide recommended therapy for such patients without postponing care.

## Conclusions

In our case, a young girl with atypical symptoms of JPM was suffering from several months of chronic muscle weakness and presented with an acute episode of generalized body swelling and difficulty swallowing. The delayed diagnosis of her condition led to several complications and ultimately resulted in her demise. Despite JPM’s rarity in the pediatric population, the clinician should have a high level of clinical suspicion if a young child presents with generalized body swelling, dysphagia, and proximal muscle weakness. Autoimmune conditions with atypical presentations sometimes go undiagnosed for long periods of time, which can have devastating consequences if not addressed promptly. Early use of immunosuppressive and steroid medications may help reduce the complications.
